# Association between processed and unprocessed red meat consumption and risk of nonalcoholic fatty liver disease: A systematic review and dose-response meta-analysis

**DOI:** 10.7189/jogh.14.04060

**Published:** 2024-04-26

**Authors:** Qin Zhou, Huaidong Hu, Lina Hu, Shuaibin Liu, Jin Chen, Shiwen Tong

**Affiliations:** 1 Department of Clinical Nutrition, The Second Affiliated Hospital of Chongqing Medical University, Chongqing, China; 2 Department of Endocrinology and Metabolism, Chongqing General Hospital, Chongqing, China; 3 Department of Obstetrics and Gynecology, The Second Affiliated Hospital of Chongqing Medical University, Chongqing, China; 4 Department of Evidence-based Medicine and Clinical Epidemiology, West China Medical School of Medicine/West China Hospital, Sichuan University, Chengdu, China

## Abstract

**Background:**

The nature of the relationship between red meat consumption and nonalcoholic fatty liver disease (NAFLD) remains unclear. Through this meta-analysis, we aimed to determine the association and dose-response relationship between red meat consumption (both processed and unprocessed) and the risk of NAFLD.

**Methods:**

We systematically searched CENTRAL, PubMed, Embase, Web of Science and Scopus from inception to February 2022 for observational studies in which the exposure of interest was red meat consumption; the outcome of interest was the risk of NAFLD; and where odds ratios (ORs) or risk ratios were provided or could be calculated. We used random-effects meta-analyses to pool the effect sizes and performed analyses to estimate the linearity of the dose-response relationships between red meat intake and NAFLD risk.

**Results:**

We included 10 studies in this review. The meta-analysis showed a significant association between the intake of red meat (OR = 1.27; 95% confidence interval (CI) = 1.07–1.50, *P* = 0.000, *I*^2^ = 81%), processed red meat (OR = 1.20; 95% CI = 1.04–1.3, *P* = 0.162, *I*^2^ = 34.9%) or unprocessed red meat (OR = 1.28; 95% CI = 1.05–1.55, *P* = 0.001, *I*^2^ = 76.2%) and the risk of NAFLD. We also found a significant linear dose-response association between processed red meat intake and NAFLD, with each 25-g increment of processed red meat intake per day was associated with an 11.1% higher risk of NAFLD (OR = 1.11; 95% CI = 1.01–1.22, *P* = 0.029), and a nonlinear association between unprocessed meat intake and NAFLD (*P* = 0.003 for nonlinearity).

**Conclusions:**

Our findings indicate a potential positive association between red meat consumption (both processed and unprocessed) and NAFLD risk, especially in relation to increased intake of processed red meat compared to unprocessed red meat. However, caution is advised in interpreting these results; further research could establish a clearer understanding of the relationship between red meat consumption and NAFLD risk.

**Registration:**

PROSPERO: CRD42022332839.

Nonalcoholic fatty liver disease (NAFLD), which refers to a wide spectrum of liver damage ranging from simple steatosis to steatohepatitis, advanced fibrosis, and cirrhosis [1], has emerged as a prominent cause of liver disease worldwide during the last two decades, with prevalence ranging from 25% to 45% [2]. Moreover, around 25% to 44% of people with simple steatosis will progress to steatohepatitis in three to six years, which can further develop to cirrhosis, hepatocellular carcinoma, and end-stage liver disease [3]. Because of the high number of NAFLD cases, nonalcoholic steatohepatitis has become the second most common and the fastest-growing indication for liver transplantation [4]. Furthermore, Simon et al. [5] found that even with mild steatosis, the risk of all-cause mortality increased by 71%. NAFLD, therefore, is becoming a more significant public health challenge, especially due to the growing epidemics of obesity and diabetes [6].

Although its aetiology is not fully understood, it is generally believed that harmful dietary components may predispose individuals to NAFLD [7]. Epidemiological studies and meta-analyses have shown that a high intake of red meat increases the risk of several chronic diseases, especially type 2 diabetes, and colorectal cancer [8]. There is also evidence that people with NAFLD are more inclined to overeat red meat and its products [9]. This is especially concerning as red meat consumption has increased globally in recent decades, particularly in underdeveloped countries [10].

Research has suggested that a higher intake of red meat is associated with an increased risk of NAFLD [11–14]; however, Kim et al. [15] and Honarvar et al. [16] found no such link. Some studies examined the relationship between the consumption of processed and unprocessed red meat and the risk of developing NAFLD independently, but again with inconsistent findings [11–14,17]. Our study is the first systematic review and dose-response meta-analysis to evaluate the association between red meat and its specific subtypes with the risk of NAFLD. With this research, we sought to provide evidence for public dietary guidance on red meat.

## METHODS

We followed the PRISMA 2020 guidelines released in 2020 in reporting our findings [18]. The protocol registration is available in PROSPERO (CRD42022332839).

### Search strategy

We systematically searched CENTRAL, PubMed, Embase, Web of Science and Scopus from inception to 10 February 2022, without restrictions to language or year of publication. The search strings comprised a combination of MeSH and non-MeSH terms with keywords related to the dietary intake of red meat and the risk of NAFLD (**Online Supplementary Document**). We also conducted a web-based search in Google Scholar using a combination of terms related to ‘red meat’ and ‘non-alcoholic fatty liver disease’, screening the first 500 results as ranked by relevancy. We finally checked the reference lists of the selected papers and relevant reviews for any works that may have been missed.

### Inclusion and exclusion criteria

Two authors (QZ and HDH) separately screened titles and abstracts for relevance, resolving discrepancies through discussion with a third researcher (JC). We included observational studies (eg, cohort, case-control, nest case-control, and cross-sectional studies) which included apparently healthy adults (age ≥18 years) and assessed the association between the consumption of red meat as exposure and the risk of NAFLD as an outcome, while reporting either relative risks (RRs) or odds ratios (ORs) with corresponding 95% confidence intervals (CIs). If the findings of a study were published in more than one article, we chose the most recent publication; otherwise, we selected the study with the most cases or the highest quality. We excluded letters, commentaries, meeting abstracts, conference papers, reviews, editorials, and ecological studies, as they are too brief and usually present only viewpoints or commentaries. We also excluded the following studies: Those involving pregnant or lactating women; cohort studies with participants diagnosed with liver diseases at baseline, such as fatty liver disease, NASH, chronic hepatitis B or C, autoimmune liver disease, and similar conditions; studies only assessing the combined consumption of red meat, poultry (such as chicken), or fish; and studies lacking available full-text access.

### Data extraction

Three reviewers (QZ, HDH, and JC) extracted data from the included studies using a standardised data collection form. This included the first author’s last name; year of publication; study design; sample size; number of cases; participant characteristics (age, gender); study location; follow-up years; methods used to assess dietary intake of red meat; red meat intake (quantitatively measured by gram or by serving); relevant effect sizes of comparison categories, together with 95% CIs; and confounding variables which were adjusted for in the statistical analysis. Any disagreements in data extraction were solved by consensus after discussion with a fourth reviewer (JC). When results of a study for men and women were reported separately, we treated each analysis as a separate study.

### Risk of bias assessment

Two researchers (QZ and SBL) evaluated the methodological quality of the studies using the Newcastle-Ottawa Quality Assessment Scale (NOS), developed for evaluating the quality of nonrandomised studies in meta-analyses [19]. The scale sets a maximum score of eight or nine points for studies exhibiting the lowest bias across three primary categories: Study group selection (four points); study group comparability (two points); and exposure and outcome ascertainment (two or three points). Exposure and outcome ascertainment assigns three points for case-control studies, three points for cohort studies, and two points for cross-sectional studies, respectively. We judged studies that received a score of eight or nine points to be at low risk of bias, studies that scored six or seven points to be at medium risk of bias, and those that scored less than six points to be at high risk of bias. In case of disagreement, a third reviewer (JC) was invited for consultation.

### Statistical analysis

We assumed all ORs and their respective 95% CIs as the effect size, transforming all RRs or hazard ratios (HRs) to ORs to be merged. We assessed for heterogeneity among the studies using Cochran’s Q test, with *P* < 0.10 indicating statistically significant heterogeneity, and the *I*^2^ statistics, with values of 25%, 25–50%, 50–75%, and 75% categorised as no, modest, moderate, and significant heterogeneity, respectively. We deemed inter-study heterogeneity to exist when *I*^2^ exceeded 50% or the *P*-value for Cochran’s Q was <0.1. Due to the observed heterogeneity among studies, we chose the DerSimonian and Laird random-effects model [20] for our meta-analysis. This model, known for providing more conservative estimates in the presence of substantial variability, is particularly suitable when effects vary both within and between studies. We therefore used it to compute pooled effect sizes, with weights generated through the inverse variance method. This approach accounts for the precision of each study, assigning greater weight to those with smaller variances, resulting in a nuanced and conservative overall effect size estimation.

We also performed a subgroup analysis to investigate the effect of research design, study quality (high, middle, or low), body mass index (BMI) (yes or no), smoking (yes or no), physical activity (yes or no), and calorie intake (yes or no) on the association between exposures and outcomes. We used the trim and fill method to account for publication bias in our meta-analyses [21]; Egger's regression asymmetry test [22] to statistically determine funnel plot asymmetry; and sensitivity analyses through the leave-one-out method to examine the potential impact of each study on the overall estimate.

We conducted our dose-response meta-analyses according to the method introduced by Greenland and Longnecker [23] and Orsini, Bellocco, and Greenland [24]. This method estimates slope lines for each study and combines them to obtain an overall average slope. A random-effects model was used to merge study-specific slope lines. In this method, studies should cover more than two categories of red meat consumption, and the number of cases and/or person-years; median point or mean of red meat intake in each category; and the odds ratios, relative risks, or hazard ratios with variance estimates should be reported. We used the category midpoints in cases where red meat intake was only reported as a range. When the highest or lowest intake categories was unbounded, we estimated the median point by multiplying the top end by 1.5 and dividing the lower end by 1.5. For studies that reported meat intake as serving or time, we used the serving sizes according to prior studies [15], which were 50 g of total red meat; 35 g of processed meat; and 85 g of unprocessed red meat. We computed daily red meat intake using a mean energy intake of 2000 kcal per day for studies that reported in grams per 1000 kcal.

We also explored a potential nonlinear connection between red meat intake and the prevalence of NAFLD. We modelled the red meat intake by conducting restricted cubic splines with three knots at fixed centiles of 10%, 50%, and 90% of the distribution. We took into account the correlation within each group of risk estimations and used a one-stage weighted mixed-effects meta-analysis [25] to combine the study-specific estimates. We determined the significance of nonlinearity using null hypothesis testing, with the coefficient of the second spline set to zero. We conducted the dose-response analysis on total red meat, processed red meat, unprocessed meat separately. All statistical analysis were conducted in Stata, version 15 (Stata Corp LLC, College station, TX, USA).

### Definition of red meat, processed red meat and unprocessed red meat

We noted that the terms ‘unprocessed meat’ and ‘processed meat’ were not consistently defined in the included studies. Here we uniformed the definitions of red meat, processed red meat, and unprocessed red meat per the International Agency for Research on Cancer (IARC) definitions [26] (Table S1 in the **Online Supplementary Document**). Unprocessed red meat included the unprocessed muscle meat of mammals, such as beef, pork, mutton, veal, horse, lamb, or goat, usually in minced or frozen form and eaten cooked. Processed red meat referred to red meat that has been processed by salting, curing, fermentation, smoking, or other processes that enhance the flavour or improve preservation (eg, bacon or ham), and often containing high quantities of minced fatty tissues (eg, sausages). Red meat was the generic term for both unprocessed red meat and processed red meat.

## RESULTS

### Study selection

**Figure 1:**
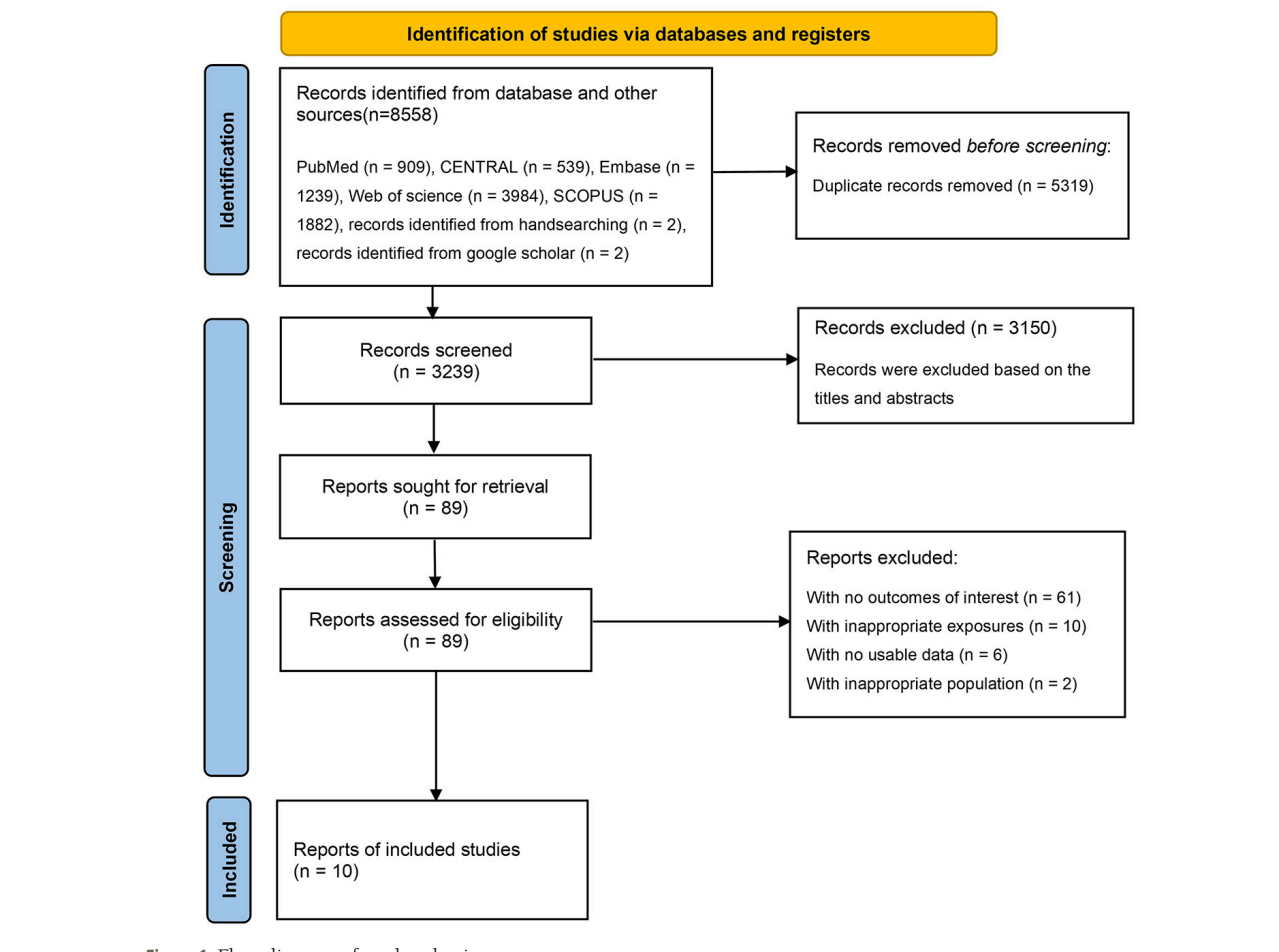
Flow diagram of study selection.

We identified 8558 articles in the initial search. Following the exclusion of duplicates (n = 5319) and studies found to be ineligible during the title/abstract screening (n = 3150), we assessed the full text of 89 possibly relevant articles (**Figure 1**). Finally, we included 10 observational studies in the analysis. Eight studies assessed the association between red meat and NAFLD [11–16,27,28], seven described the relationship between processed red meat and NAFLD [11–13,15–17,27], and five investigated the link between unprocessed red meat and NAFLD [11,14–17,29].

### Characteristics of included studies

The included studies were published between 2017 and 2022; used a cohort (n = 3) [11,14,15], cross-sectional studies (n = 5) [12,13,16,27,29], and case-control design (n = 2) [17,28], and enrolled 119 343 participants (range: 210–77 795) aged 25–75 years, with 8816 cases (**Table 1**). The follow-up periods for the cohort studies ranged from seven to more than 20 years. Regarding specificities in study populations, Kim et al. [15] included only women, while Zhou et al. [29] reported data for men and women separately, so we treated this article as two separate analyses. Two studies were conducted in the USA [11,15], while the remaining ones were reported from Asian countries like China [12,29], Iran [16,17,28], Israel [13], and Thailand [27]. Eight studies used food frequency questionnaires (FFQs) [11–17,29] to assess dietary red meat intake, one used three-day food diaries [28], and one used photographs and seven-day food diaries [27]. NAFLD was diagnosed by abdominal ultrasonography or computed tomography scanning in eight study [11–14,16,17,28,29], by physician-confirmed diagnosis in one study [15] and by transient elastography and controlled attenuation parameter (CAP) in one study [27]. Most included studies had controlled some important confounders like age (n = 6) [13,14,16,17,27,29], BMI (n = 6) [11,13,15–17,28,29], physical activity(n = 8) [11–15,17,28,29], energy intake (n = 8) [11–17,27], and alcohol consumption (n = 4) [11,13,14,29]. Overall, two studies were of high quality [11,15], four were of middle quality [13,14,16,29] and four were of low quality [12,17,27,28] (Table S2 in the **Online Supplementary Document**).

**Table 1:** Characteristics of included studies

Author (year)	Study design	Country	Age year	Follow-up, year	Sample size	NAFLD, n	Female, %	Exposure assessment	Exposure	Median/cutoff point	OR (95%)	Adjustment
Kim et al. (2021) [15]	Cohort	USA	25–42	10	77 795	3130	81	FFQ	Total red meat	≤1 serving/w, 2–4 servings/w, 5–6 servings/w, 1 serving/d, ≥2 servings/d, per 1 serving/d increase	1, 1.02 (0.82–1.27), 0.995 (0.81–1.23), 0.995 (0.80–1.24), 0.999 (0.81–1.24), 0.99(0.89–1.10)	Adjusted for total caloric intake, diabetes, hypertension, dyslipidaemia, smoking status, physical activity, regular use of aspirin use, menopausal status, and menopausal hormone use, BMI.
									Processed red meat	<1 serving/mo, 1–3 servings/mo, 1 servings/w, 2–4 serving/w, ≥5 servings/w, per 1 serving/d increase	1, 1.01 (0.82–1.24), 0.99 (0.81–1.21), 1.04 (0.86–1.27), 1.06 (0.85–1.31), 1.12 (0.89–1.41)	
									Unprocessed red meat	≤1 serving/w, 2–4 servings/w, 5–6 servings/w, 1–2 serving/d, ≥2 servings/d, per 1 serving/d increase	1, 1.12 (0.94–1.34), 1.02 (0.85–1.21), 1.09 (0.90–1.32), 1.02 (0.83–1.25), 0.95 (0.83–1.09)	
Hashemian et al. (2021) [14]	Cohort	Iran	40–75	7	1612	505	48.4	FFQ	Total red meat	4g/d, 10g/d, 18g/d, 34g/d	1, 1.17 (0.78–1.77), 1.70 (1.15–2.53), 1.59 (1.06–2.38)	Adjusted for age, sex, WC, formal education, smoking status, opium use, physical activity, ethnicity, wealth score, alcohol drinking, and total energy intake.
									Processed red meat	0g/d, 1g/d, 5g/d	1, 1.03 (0.77–1.38), 1.08 (0.81–1.46)	
									Unprocessed red meat	2g/d, 6g/d, 12g/d, 25g/d	1, 1.66 (1.07–2.59), 1.81 (1.17–2.79), 1.73 (1.13–2.66)	
Noureddin et al. (2020) [11]	Cohort	USA	x̄ = 57.7 (SD = 7.8)	>20	32448	2974	63	FFQ	Total red meat	≤13.7g/d, 13.7g/d –23.3g/d, 23.3g/d –34.0g/d, >34.0g/d	1, 1.08 (0.96–1.21), 1.12 (1.00 –1.26), 1.15 (1.02–1.29)	Adjusted for BMI, alcohol intake, coffee, total soda consumption, vigorous physical activity, and energy.
									Processed red meat	≤3.0g/d, 3.0g/d –6.1g/d, 6.1g/d–10.0g/d, >10.0g/d	1, 1.03 (0.92–1.16), 1.05 (0.94–1.18), 1.18 (1.05–1.32)	
									Unprocessed red meat	≤9.3g/d, 9.3g/d –16.2g/d, 16.2g/d –24.1g/d, >24.1g/d	1, 1.08 (0.97–1.21, 1.11 (0.99–1.24), 1.16 (1.04–1.30)	
Charatcharoenwitthaya et al (2021) [27]	Cross–sectional	Thailand	x̄ = 37.6 (SD = 10.0)	NA	252	41	81	Took photographs and food diary	Total red meat	≥50g/d, <50g/d	1, 1.09 (0.52–2.27)	Adjusted for age, sex, health care profession, and daily calorie intake.
Peng et al. (2021) [12]	Cross–sectional	China	x̄ = 53.54 (SD = 6.90)	NA	1594	532	46.5	FFQ	Total red meat	<28.44g/d, 28.44–49.74g/d, 49.75–71g/d, >71g/d per 50g/d increase	1, 1.948 (1.399–2.741), 1.190 (0.833–1.698), 1.716 (1.214–2.424), 1.143 (1.010–1.294)	Adjusted for smoking, tea intake, weekly hours of physical activity and presence of hypertension, dyslipidaemia and diabetes, energy and cholesterol intake.
									Processed red meat	<2.26g/d, 2.26–4.61g/d, 4.62–6.59g/d, >6.59g/d, per 50g/d increase	1, 1.46 (0.806–1.629), 1.389 (0.992–1.946), 1.335 (0.938–1.901), 0.965 (0.766–1.216)	
Zelber-Sagi et al. (2018) [13]	Cross–sectional	Israel	x̄ = 58.83 (SD = 6.58)	NA	789	305	47.4	FFQ	Total red meat	Divided above and below the population’s median consumption	1, 1.47 (1.04–2.09)	Adjusted for age, gender, energy intake per day and BMI. physical activity, smoking status, alcohol drinking, saturated fat, and cholesterol intake.
									Processed red meat		1, 1.20 (0.85–1.68)	
Zhou et al. (2019) [29]	Cross–sectional	China	25–74	NA	3166	716	60.9	FFQ	Unprocessed red meat	≥200g/w vs <200g/w(women), ≥325g/w vs <325g/w(men)	1, 1.41 (1.09–1.81), 1.00 (0.76–1.33)	Adjusted for age, BMI, systolic blood pressure, HbA1c, total cholesterol, triglycerides, ALT and uric acid, post–menopausal status (for women), annual house income, education level, physical activity level, smoking status (for men), red meat intake, and alcohol drinking.
Honarvar et al. (2017) [16]	Cross–sectional	Iran	x̄ = 42 (SD = 31.51)	NA	478	204	57.5	FFQ	Total red meat	NR	1, 0.99 (0.98–1.02)	Adjusted for age, gender, BMI, marital statu, and energy intake.
Tutunchi et al. (2021) [28]	Case–control	Iran	x̄ = 45.5 (SD = 9.17)	NA	210	105	57.2	A three–day food diary	Total red meat	Lowest tertile, top tertile	1, 2.68 (1.31–4.16)	Adjusted for sex, education, physical activity, BMI, and WC.
Rahimi-Sakak et al. (2022) [17]	Case–control	Iran	x̄ = 45.54 (SD = 14.13)	NA	999	196	56.96	FFQ	Processed red meat	<0.36g/d, 0.38–2.38g/d, 2.38–6.58g/d, >6.58g/d	1, 1.72 (0.84–3.52), 2.36 (1.19–4.65), 3.25 (1.57–6.73)	Adjusted for age and gender, BMI, energy intake, dietary factors, diabetes, smoking, and physical activity.
									Unprocessed red meat	<15.2g/d, 15.2–28g/d, 28–43.7g/d, >43.7g/d	1, 1.04 (0.49–2.20), 1.41 (0.69–2.91), 3.65 (1.85–7.18)	

ALT –, BMI – body mass index, CI – confidence interval, d – day, FFQ – food frequency questionnaire, NA – not applicable, NAFLD – nonalcoholic fatty liver disease, NR – not reported, OR – odds ratio, SD – standard deviation, w – week, WC – waist circumference, x̄ – mean

### Red meat and risk of NAFLD

**Figure 2:**
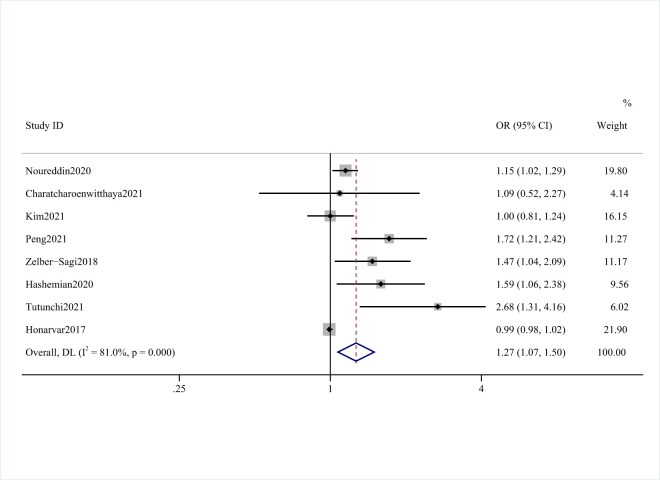
Forest plot of the risk of NAFLD and red meat consumption.

Eight studies [11–16,27,28] involving 115 178 participants and 7904 NAFLD cases investigated the association of red meat consumption and NAFLD. Five studies [11–14,28] reported a positive association, while the remaining three found no significant relationship [15,16,27]. In comparing the highest intake of red meat categories with the lowest, we found a significant positive association between red meat intake with the risk of NAFLD (OR = 1.27; 95% CI = 1.07–1.50, *P* = 0.000; *I*^2^ = 81.0%) (**Figure 2** and **Table 2**).

**Table 2:** Subgroup analysis and meta-regression between red meat, processed red meat, and unprocessed red meat intake and NAFLD risk

				*P*-value	
**Highest vs lowest red meat intake**	**Number of effect sizes**	**Pooled OR (95% CI)**	***I*^2^ (%)**	**Heterogeneity**	**Meta-regression**
Red meat					
*Overall*	8	1.27 (1.07–1.50)*	81.0	0.000	
Study location					0.252
*USA*	2	1.10 (0.97–1.25)	22.3	0.257	
*Asian countries*	6	1.47 (1.06–2.04)*	83.9	0.000	
Sex					0.333
*Men and women*	7	1.34(1.10–1.64)*	83.7	0.000	
*Women*	1	1.00 (0.81–1.24)			
Adjustment for BMI					0.165
*Yes*	3	1.06 (0.90–1.25)	59.3	0.086	
*No*	5	1.52 (1.12–2.05)*	70.0	0.01	
Study design					0.202
*Cross-sectional study*	4	1.28 (0.91–1.79)	79.5	0.002	
*Cohort study*	3	1.15 (0.96–1.38)	51.2	0.129	
*Case-control study*	1	2.68 (1.50–4.78)*			
Study quality					0.225
*High quality*	2	1.10 (0.97–1.25)	22.3	0.257	
*Middle quality*	3	1.27 (0.89–1.80)	80.3	0.006	
*Low quality*	3	1.78 (1.17–2.69)*	45.5	0.159	
Adjustment for energy					0.067
*Yes*	7	1.19 (1.02–1.38)*	76.5	0.000	
*No*	1	2.68 (1.50–4.78)*			
Adjustment for physical activity					0.960
*Yes*	5	1.29 (1.07–1.55)*	62.3	0.031	
*No*	3	1.39 (0.73–2.63)	82.6	0.003	
Adjustment for smoking					0.084
*Yes*	3	1.59 (1.29–1.96)*	0	0.862	
*No*	5	1.11 (0.95–1.30)	77.1	0.002	
Processed red meat					
*Overall*	7	1.20 (1.04–1.37)*	34.9	0.162	
Study location					0.555
*USA*	2	1.15 (1.04–1.28)*	0	0.390	
*Asian countries*	5	1.32 (1.01–1.73)*	48.7	0.099	
Sex					0.452
*Men and women*	6	1.24 (1.05–1.47)*	38.5	0.149	
*Women*	1	1.06 (0.85–1.32)			
Adjustment for BMI					0.964
*Yes*	3	1.40 (0.90–2.19)	76.2	0.015	
*No*	4	1.18 (1.07–1.30)*	0	0.845	
Study design					0.110
*Cross-sectional study*	3	1.25 (0.99–1.58)	0	0.893	
*Cohort study*	3	1.14 (1.04–1.26)*	0	0.636	
*Case-control study*	1	3.25 (1.57–6.73)*			
Study quality					0.448
*High quality*	2	1.15 (1.04–1.28)*	0	0.390	
*Middle quality*	2	1.13 (0.90–1.41)	0	0.647	
*Low quality*	3	1.65 (0.95–2.87)	61.7	0.074	
Adjustment for energy					0.978
*Yes*	7	1.20 (1.04–1.37)*	34.9	0.162	
*No*	0				
Adjustment for physical activity					0.541
*Yes*	6	1.20 (0.55– 2.43)	45.7	0.101	
*No*	1	1.16 (0.55–2.43)			
Adjustment for smoking					
*Yes*	4	1.36 (1.00–1.86)	61.3	0.052	
*No*	3	1.15 (1.04–1.27)*	0	0.691	
Unprocessed red meat					
*Overall*	6	1.28 (1.05–1.55)*	76.2	0.001	
Study location					0.199
*USA*	2	1.09 (0.97–1.24)	51.2	0.152	
*Iran*	2	2.39 (1.16–4.95)*	70.0	0.068	
*China*	2	1.19 (0.85–1.67)	68.2	0.076	
Sex					0.540
*Men and women*	3	1.79 (1.01–3.17)*	85	0.001	
*Women*	2	1.18 (0.86–1.61)	79.1	0.029	
*Men*	1	1.00 (0.76–1.32)			
Adjustment for BMI					0.995
*Yes*	4	1.32 (0.95–1.83)	82.6	0.001	
*No*	2	1.34 (0.92–1.95)	68.1	0.077	
Study design					0.177
*Cohort study*	3	1.16 (0.97–1.38)	67.5	0.046	
*Case-control study*	1	3.65 (1.85–7.19)*			
*Cross-sectional study*	2	1.19 (0.85–1.67)	68.2	0.076	
Study quality					0.137
*High quality*	2	1.09 (0.97–1.24)	51.2	0.152	
*Middle quality*	3	1.31 (0.97–1.77)	63.2	0.066	
*Low quality*	1	3.65 (1.85–7.19)*			
Adjustment for energy					
*Yes*	6	1.28 (1.05–1.83)*	82.6	0.001	
*No*	0				
Adjustment for physical activity					
*Yes*	6	1.28 (1.05–1.83)*	82.6	0.001	
*No*	0				
Adjustment for smoking					0.344
*Yes*	2	1.09 (0.97–1.24)	51.2	0.152	
*No*	4	1.58 (1.06–2.35)*	78.7	0.003	

BMI – body mass index; CI – confidence interval, OR – odds ratio

*Statistically significant.

We evaluated the potential dose-response associations among red meat intake and risk of NAFLD based on four studies that provided sufficient data for analysis [11,12,14,15]. The median red meat intake categories ranged from 4 to 195 g/d. We found no evidence of a linear (OR = 1.00; 95% CI = 0.93–1.09; *P* = 0.007, *I*^2^ = 27.4) or nonlinear association (*P* = 0.336) between red meat intake and risk of NAFLD.

### Processed red meat and risk of NAFLD

**Figure 3:**
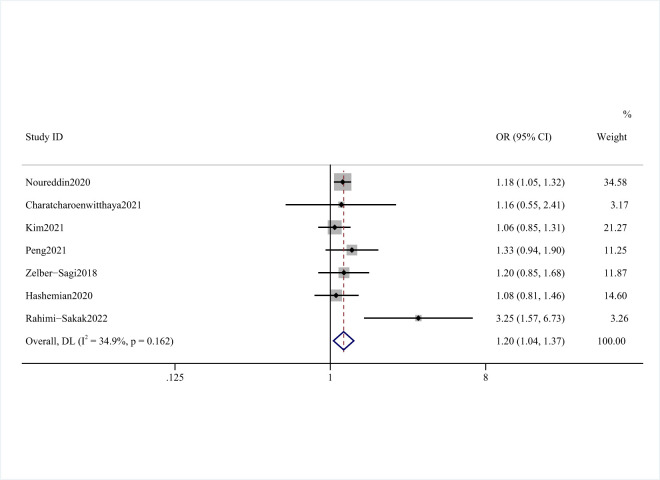
Forest plot of the risk of NAFLD and processed red meat consumption.

Seven studies [11–15,17,27] with 115 489 participants and 7683 NAFLD cases investigated the association between processed red meat and NAFLD. Two found a significant association [11,17] and five observed no significant relationship [12–15,27]. After combining the data from these studies and comparing the highest intakes of processed meat with the lowest, we observed a significant positive association between processed red meat consumption and the risk of NAFLD (OR = 1.20; 95% CI = 1.04–1.37, *P* = 0.162, *I*^2^ = 34.9%) (**Figure 3** and **Table 2**).

**Figure 4:**
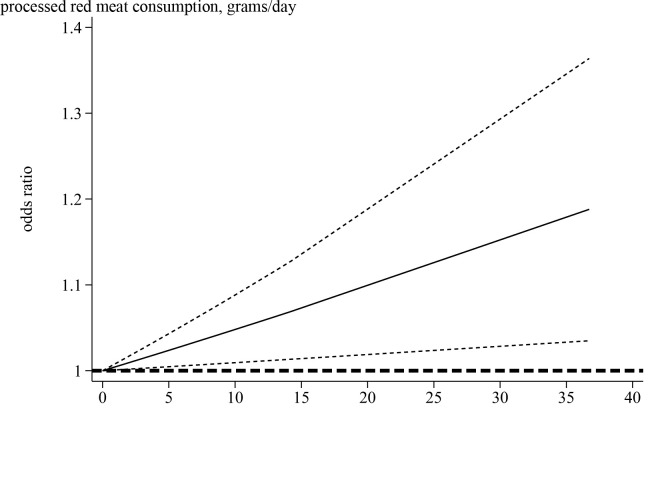
Linear dose-response relation between processed red meat intake and risk of NAFLD.

Five studies [11,12,14,15,17] with sufficient data were eligible for the analysis of the potential dose-response associations among processed red meat intake and risk of NAFLD. The median processed red meat intake categories ranged from 0 to 37.5 g/d. There was a linear relationship between processed red meat intake and risk of NAFLD. Each 25 g processed red meat intake per day was associated with a 11% higher risk of NAFLD (OR = 1.11; 95% CI = 1.01–1.22, *P* = 0.029) (**Figure 4**). However, we did not find a nonlinear relationship (*P* = 0.12).

### Unprocessed red meat and risk of NAFLD

**Figure 5:**
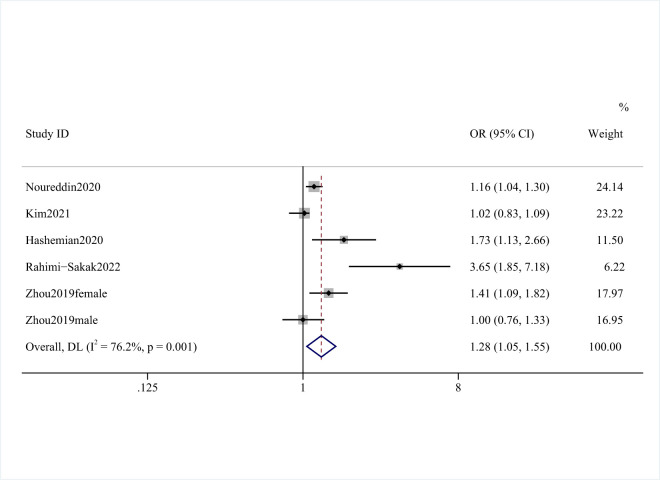
Forest plot of the risk of NAFLD and unprocessed red meat consumption.

There were five studies (six analyses) [11,14,15,17,29] on the association between unprocessed red meat and NAFLD, with 116 020 participants and 7521 NAFLD cases. Three studies [11,14,17] found a significant association between unprocessed meat with the incident with NAFLD, while Kim et al. [15] did not. However, Zhou et al. [29] only found a significant relationship in females, but not in males. By comparing the highest categories of dietary unprocessed red meat intake with the lowest, we observed that unprocessed red meat consumption was related to a higher risk of NAFLD (OR = 1.28; 95% CI = 1.05–1.55, *P* = 0.001; *I*^2^ = 76.2%) (**Figure 5** and **Table 2**).

**Figure 6:**
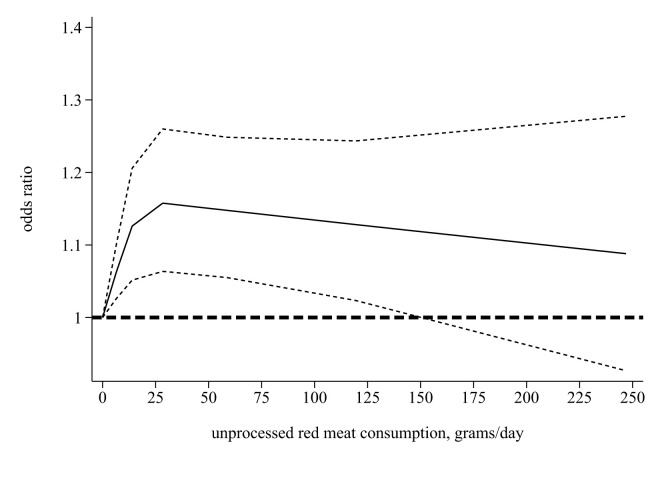
Nonlinear dose-response relation between unprocessed red meat intake and risk of NAFLD.

Four studies [11,14,15,17] were included in the analyses on the dose-response relationship between unprocessed meat and the incidence of NAFLD. The median unprocessed red meat intake categories ranged from 2 to 127 g/d. We detected no significant association in the linear dose-response analysis (OR = 1.00; 95% CI = 0.99–1.03, *P* = 0.35, *I*^2^ = 13.23%), but did find a nonlinear relationship (*P* = 0.003) (**Figure 6**). When the intake is below approximately 25 g, the risk of NAFLD rises with higher unprocessed red meat consumption, but beyond that threshold, the risk does not further increase with greater unprocessed red meat intake.

### Subgroup analyses, meta-regression, sensitivity analyses, and publication bias

We did not identify the primary variables for study heterogeneity in the meta-regression. Based on subgroup analyses, we observed a significant positive association between red meat intake and NAFLD risk in several subgroups (**Table 2**).

In the sensitivity analyses for the meta-analyses on processed red meat, the exclusion of the study by Noureddin et al. [11] showed the possibility of shifting the observed statistical significance from significant to non-significant (OR = 1.23; 95% CI = 1.00–1.51). We found a similar impact in the sensitivity analyses for unprocessed red meat for the study by Hashemian et al. (OR = 1.22, 95% CI = 1.00–1.49) [14] (**Table 2**; Figures S1–3 in the **Online Supplementary Document**).

In the publication bias results of the trim-and-fill analysis, all the effect size became non-significant post trim and fill, indicating the potential presence of publication bias that may compromise the reliability and stability of the meta-analysis. The findings from Egger's linear regression test only indicated significant publication bias in the association between red meat intake and NAFLD risk (*P* = 0.008) (Figures S4–6 and Table S3 in the **Online Supplementary Document**).

## DISCUSSION

The results of our meta-analysis showed that consumption of both processed and unprocessed red meat was linked to the development of NAFLD. There was also a significant linear dose-response association between processed red meat intake and NAFLD. Each 25-g increment of processed red meat intake per day was associated with a 11.1% higher risk of NAFLD. Moreover, we found a nonlinear association between unprocessed red meat intake and NAFLD risk, in which the risk of NAFLD increased with the increment of unprocessed red meat intake. The risk of NAFLD peaked at 30 g/d, increasing the risk by about 15%. These findings suggest a potential link between the consumption of processed and unprocessed red meat and an increased risk of NAFLD. Moreover, the risk appears to be more pronounced with higher consumption of processed red meat compared to unprocessed red meat.

Red meat is a major source of saturated fatty acids (SFAs) [30], which are less oxidized than unsaturated fatty acids, resulting in more dietary saturated fatty acids flowing into and accumulation in the liver [31,32]. A clinical study has also shown that a diet high in SFAs increases adipose tissue lipolysis, inducing more intrahepatic triglycerides than a diet high in simple sugars [33]. Accumulation of saturated fat in the liver may affect the structure and function of liver mitochondria, driving the progression of NAFLD [34]. Furthermore, saturated fat intake also causes insulin resistance [33], which may play a role in the pathophysiology of NAFLD by stimulating hepatic de novo lipogenesis and contributing to hepatic steatosis [35]. This hypothesis is partly supported by the rapid increase in the incidence of NAFLD in type 2 diabetes mellitus patients [36]. Besides, red meat is high in fat and protein. Furthermore, cooking red meat at high temperatures (by grilling, frying, or roasting) releases harmful compounds such as polycyclic aromatic hydrocarbons, heterocyclic amines, or advanced glycation end products, which have been shown to increase insulin resistance [13,37–39]. Moreover, higher levels of advanced glycation end products have been reported to be positively linked with the risk of NAFLD [40]. Additionally, red meat intake may alter gut microbiota [41], which may contribute to the development of NAFLD as well [42].

In our dose-response analysis, we discovered a linear relationship between processed red meat and the risk of NAFLD, but a nonlinear relationship between unprocessed red meat and the risk of NAFLD, implying that the additional components of processed red meat may play an important role in the development of NAFLD. The components of processed and unprocessed red meat differ in three aspects. First, SFA levels in processed red meat are typically higher than in unprocessed red meat. The proportion of fat in sausages, for example, often reaches 50% of weight or even more [43]. Second, during the preparation of processed red meat, more hazardous compounds are produced. For example, smoking red meat increases the production of polycyclic aromatic hydrocarbons [44]. Third, there are many non-meat substances added to processed red meat products, such as nitrates/nitrites, salts, phosphates, pigments, various additives, etc. Micha et al. [45,46] found that processed red meat contains on average 400% more sodium and 50% more nitrates per gram than unprocessed red meat. A recent meta-analysis indicated that high salt/sodium intake was associated with a 60% greater risk of NAFLD [47]. Nitrites and nitrates can be converted into nitrosamines in the body, which are related to insulin resistance and diabetes in animal studies [46,48]. It is still not known whether other additives play a role in the formation of NAFLD.

Of the five studies focussing on unprocessed red meat, two were carried out in the USA [11,15], two in Iran [14,17], and one in China (comprising two analyses) [29]. In contrast to the USA and China, the influence of unprocessed red meat on the risk of NAFLD was more pronounced in Iran. This could potentially be explained by variations in the cooking method of unprocessed red meat across these countries. For example, the kebab, commonly made from read meat (especially lamb or veal) is one of the most popular foods in the Middle East, particularly in Iran. Nearly 60% of subjects in a cross-sectional study conducted in Iran had a high propensity to intake kebabs, consuming them four times each month on average, while over 85% ate kebab with a lot of salt [49]. This suggests that a considerable portion of unprocessed red meat consumed in Iran is grilled, perhaps raising the risk of NAFLD. In the USA, unprocessed red meat is predominantly consumed in the form of mixed-meat dishes, burgers, and beef cuts excluding ground meat [50]. Conversely, in China, the consumption of unprocessed red meat is characterised by a more diverse range of cooking methods, including stir-frying, boiling, hot pot, grilling, and more. Evidence has shown that, independent of total red meat consumption, high-temperature and/or open-flame cooking of red meats may further increase diabetes risk among regular meat eaters [51]. Considering the previously discussed close correlation between diabetes and NAFLD, we can hypothesise that disparities in cooking method may contribute to variances in NAFLD risk between the three countries, especially in view of the consumption of unprocessed red meat. Cooking methods represent just one aspect of the potential diversity in red meat consumption patterns across various geographical regions and cultural backgrounds. Additionally, factors such as the accompanying foods, the use of sauces, and the overall dietary background may also influence the risk of NAFLD. This emphasises the influence of cultural/regional variations and potential genetic predispositions on associations between red meat consumption and NAFLD risk. Therefore, caution is warranted when generalising our results to different populations.

At the individual level, the substantial impact of varied lifestyles on NAFLD is evident in the literature. Research have shown that lifestyle factors, including physical activity, nutrition, and substance consumption (such as coffee, alcohol, and cigarettes) play a pivotal role in the initiation and progression of NAFLD [52]. The relationship between physical activity and NAFLD is supported by several studies, with insufficient physical activity and sedentary behaviour emerging as independent predictors of NAFLD [53]. Physical activity plays a pivotal role in weight reduction and maintenance, fostering a healthier body composition and reducing hepatic steatosis [54], and has been a fundamental preventive and treatment measure across the entire spectrum of NAFLD. Eight studies included in our analysis incorporated controls for physical activity. The consumption of red meat is situated within a broader dietary context, and its impact on NAFLD may be influenced by the individual's overall dietary patterns. Existing evidence suggests a correlation between NAFLD occurrence and dietary context, as measured by indices such as the healthy eating index [55] or dietary inflammatory index [56]. However, the studies included in our analysis overlooked the dietary context, which may compromise the observed association between red meat consumption and the risk of NAFLD.

Regarding smoking, accumulating data suggests that both passive and active tobacco exposure may be considered environmental stressors contributing to the progression of liver injury [57]. According to the meta-analysis conducted by Rezayat et al., both active and passive smoking are significantly associated with NAFLD [58]. However, only half of the studies included in our review controlled for this factor. Given its widespread prevalence, future studies should consider smoking as a confounding factor in their analyses.

A recent study has proposed an inverse association between coffee intake and the risk of NAFLD, whereby an intake level exceeding three cups per day was linked to a lower risk of NAFLD compared to consuming fewer than two cups per day [59]. Among the studies incorporated in our analysis, only one controlled for coffee consumption.

Most of the studies included in our analysis excluded individuals with heavy alcohol consumption or imposed limitations on alcohol intake as part of the inclusion criteria. Consequently, the sample for our meta-analysis predominantly comprised of non-drinkers or those with low to moderate amounts alcohol consumption. To date, several epidemiological studies have indicated that consuming alcohol in light to moderate amounts daily may have a protective effect against the development of NAFLD [60]. Therefore, studies on this topic should also control for alcohol intake in their analyses.

In summary, future research should comprehensively control for lifestyle factors that may impact the risk of NAFLD, but also for participants’ economic status, as some studies have suggested that countries with higher economic status tend to exhibit a higher prevalence of NAFLD [61].

Our study has several strengths, including a systematic and comprehensive search strategy, more targeted meta-analysis, and more rigorous data extraction process compared to previous studies. We also only included data related to red meat, but excluded data mixing red meat with other meats. Furthermore, we used fully adjusted model risk estimates from each study in our pooled analyses to reduce the potential effect of confounders. We also conducted the first analysis aimed at exploring both the linear and nonlinear dose-response relationship between processed and unprocessed meat and the risk of NAFLD.

However, our meta-analysis also has several limitations. First, the included studies were observational, preventing us from confirming causal relationships. Although the likelihood is small, it is possible that NAFLD influences people’s preferences for red meat. Moreover, although we employed risk estimates from the fully adjusted models in each study, uncontrolled or residual confounders might have influenced our outcomes, especially as nearly all the studies neglected dietary factors such as cooking methods and dietary background, potentially compromising the observed association between red meat consumption and the risk of NAFLD. Second, all studies used FFQ tables to investigate dietary intake, making retrospective bias inevitable and preventing accurate measurement of the amount of red meat intake. This limitation in the evaluation method may attenuate the true association between red meat consumption and NAFLD. Third, the overall research quality was low, and our dose-response analysis was mainly based on four or five studies that provided sufficient data, weakening the reliability of our findings. Lastly, the NOS evaluation showed methodological shortcomings in a significant portion of the included studies. The cumulative impact of these concerns could introduce bias in our findings, affecting the robustness and generalisability of our results and highlighting a need for cautious interpretation. This is further exacerbated by the observed publication bias, which may undermine the stability and reliability of the results.

Future studies should prioritise investigating the distinct impact of various red meat subtypes on the risk of NAFLD. Specifically, there is a need for accurately defining and differentiating between red meat, processed red meat, and unprocessed red meat. To enhance the precision of findings, it is crucial to control for potential confounding factors in the relationship between red meat intake and NAFLD risk, including consideration of cooking methods, overall dietary background (using some kind of dietary index), smoking, alcohol consumption, economic status, and others. Furthermore, the inclusion of large-scale, high-quality prospective studies from diverse regions is essential for a more comprehensive understanding of these associations.

## CONCLUSIONS

Our findings suggested a potential positive association between the consumption of red meat, including both processed and unprocessed types, and the risk of NAFLD. Notably, the risk of developing NAFLD appears to be increase with consumption of processed red meat compared to unprocessed red meat. However, caution is needed in interpreting these results. Further research should help further clarify relationship between red meat consumption and NAFLD risk.

## Additional material


Online Supplementary Document
