# Supplementary material for: Association between processed and unprocessed red meat consumption and risk of nonalcoholic fatty liver disease: A systematic review and dose-response meta-analysis
Source: J Glob Health. 2024 Apr 26;14:04060. doi: 10.7189/jogh.14.04060 (PMC11046257; doi:10.7189/jogh.14.04060)
Supplement: Online Supplementary Document [file jogh-14-04060-s001.pdf]

Appendix to Association between Processed and Unprocessed Red Meat Consumption and Risk of Non-alcoholic Fatty Liver Disease: A Systematic Review and Dose-response Meta-analysis

The example of search strategy:
(((“non-alcoholic” OR “nonalcoholic” OR “steatohepatitis”) AND (“fatty liver”)) OR “NAFLD” OR “non-alcoholic fatty liver disease” OR “nonalcoholic fatty liver disease” OR “NASH” OR “NAFL” OR “metabolic dysfunction-associated fatty liver disease” OR “mafld”) AND (“meat” OR “beef” OR “veal” OR “hot dog” OR “salami” OR “sausage” OR “lamb” OR “bacon” OR “deli meat” OR “pastrami” OR “hamburger” OR “schnitzel” OR “diet”).

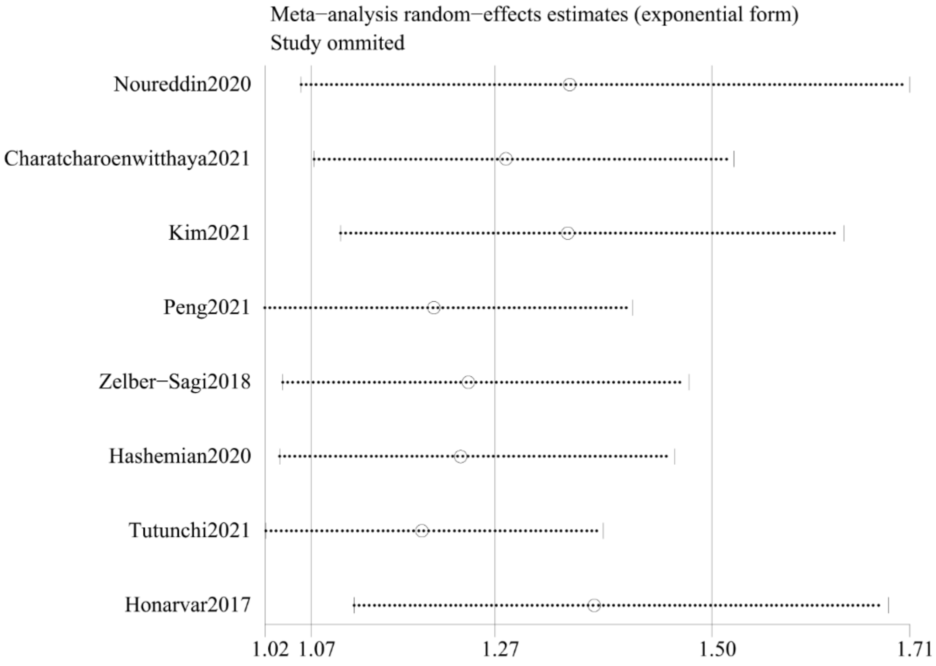

Figure S1. Sensitive analysis for red meat

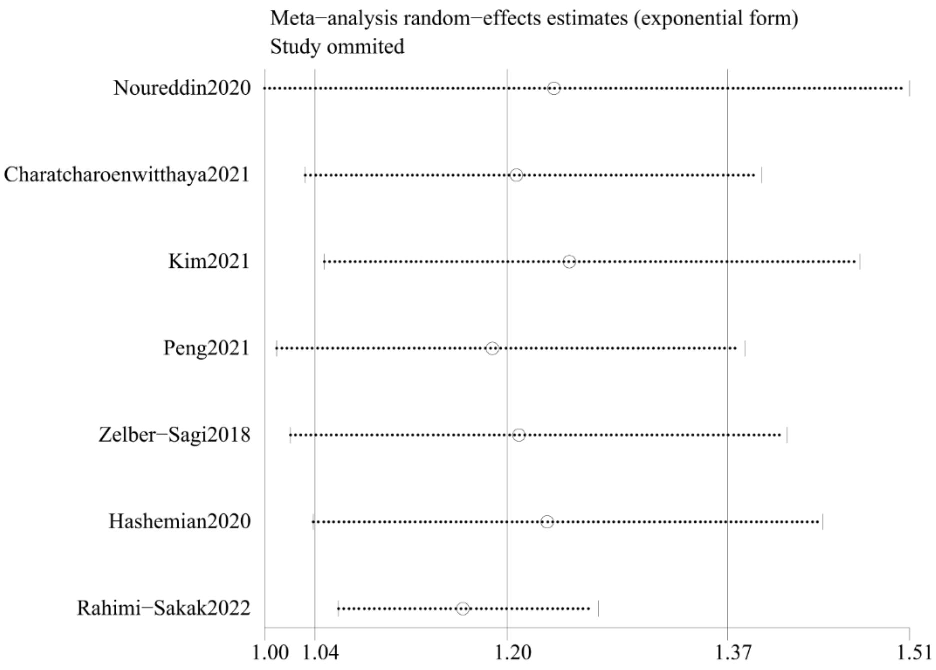

Figure S2. Sensitive analysis for processed red meat

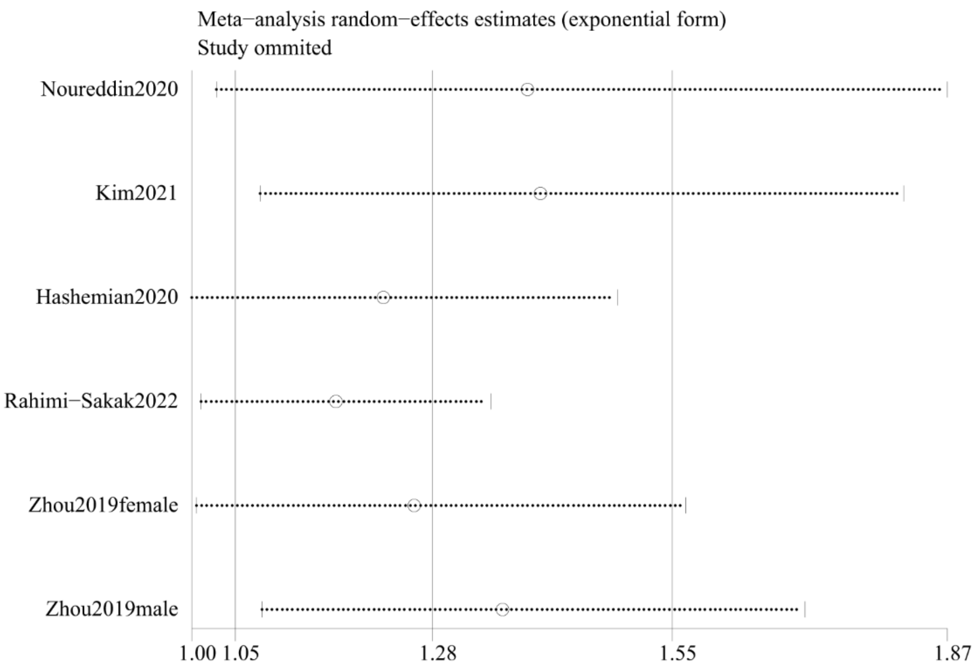

Figure S3. Sensitive analysis for unprocessed red meat

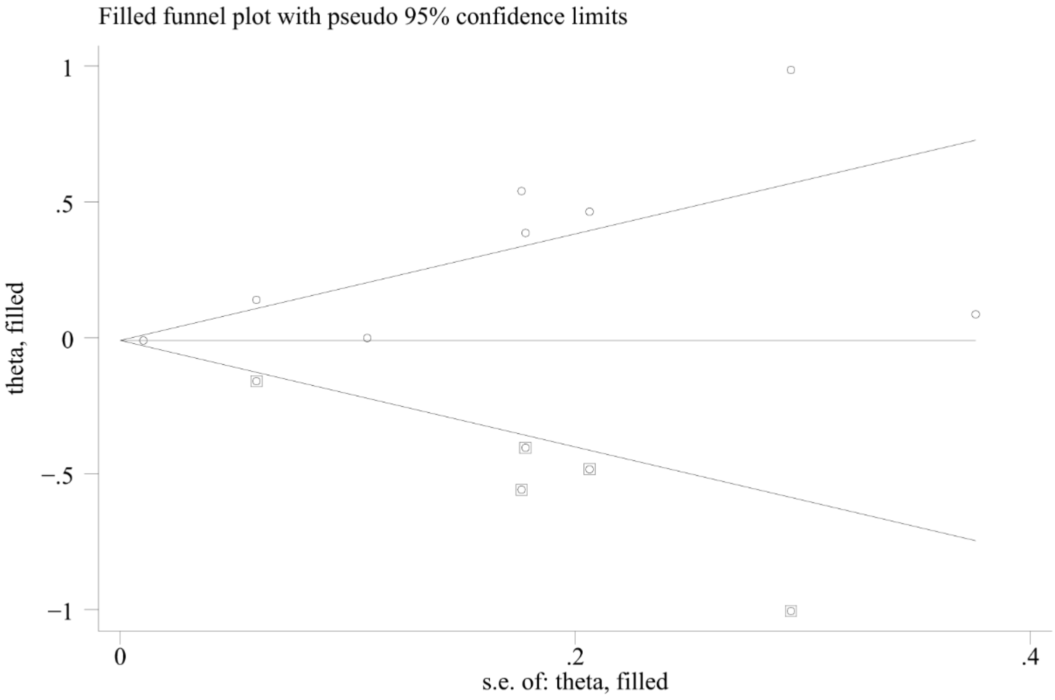

Figure S4. trim-and-fill funnel plot for red meat

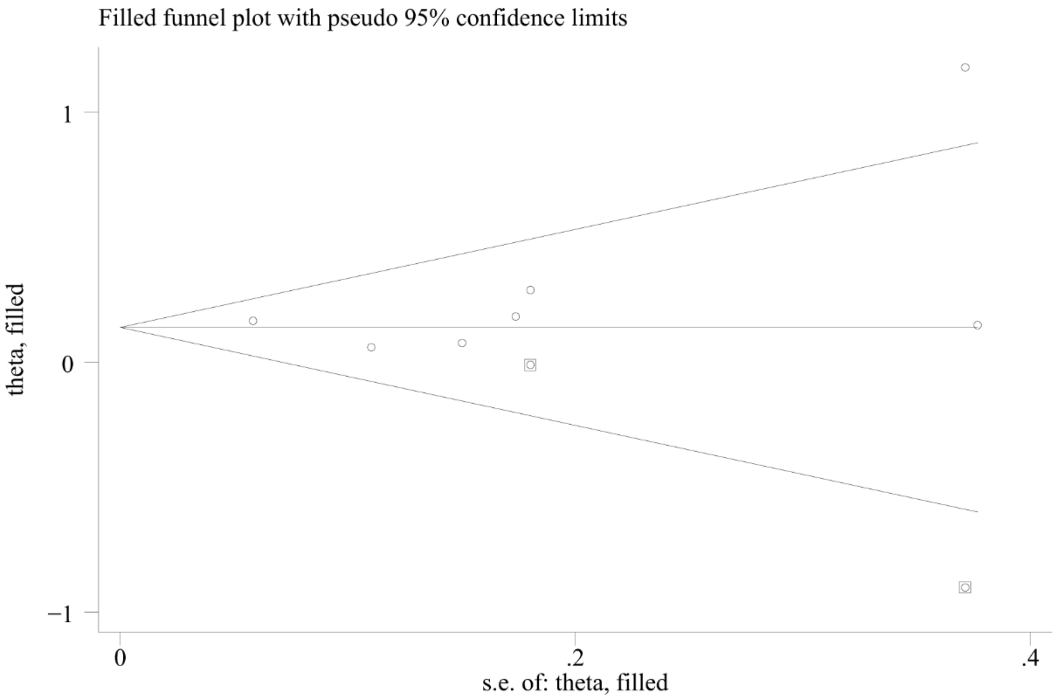

Figure S5. trim-and-fill funnel plot for processed red meat

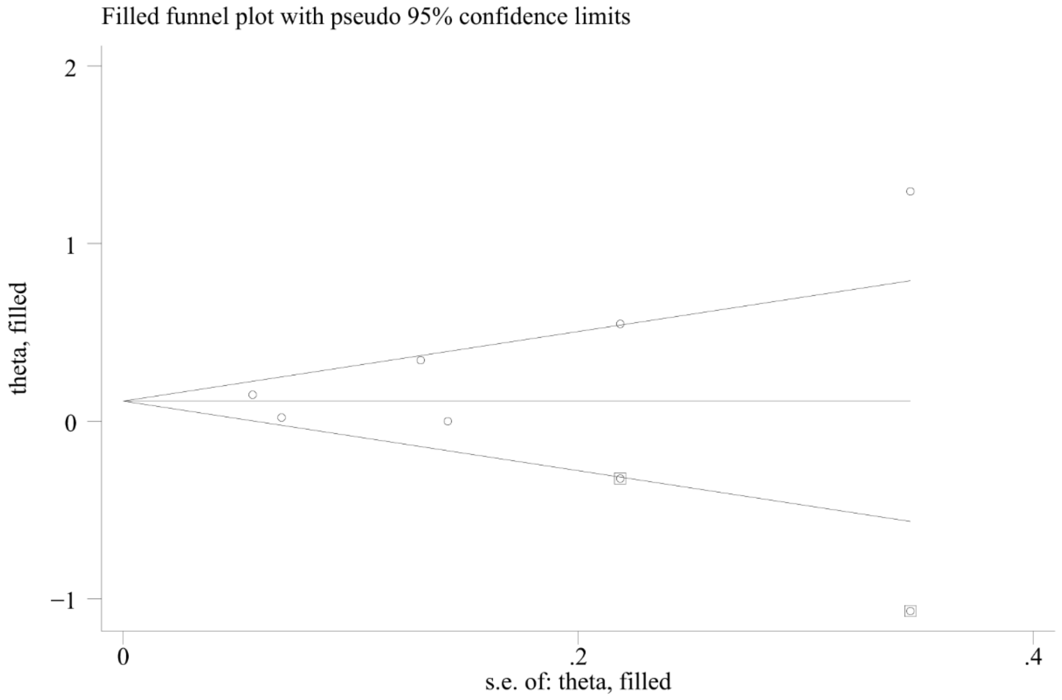

Figure S6. trim-and-fill funnel plot for unprocessed red meat

Table S1. Definitions of red meat, unprocessed red meat and processed meat used in studies included in the systematic review.

Study ID	Red meat		Unprocessed red meat		Processed red meat	
	**Terminology in the study**	**Description in the study**	**Terminology in the study**	**Description in the study**	**Terminology in the study**	**Description in the study**
Noureddin2020	Red meat	none	red meat excluding processed meat	none	Processed red meat	none
Charatcharoenwitthaya2021	Red meat	none	none	none	none	none
Kim2021	Total red meat	none	unprocessed red meat	unprocessed red meat intake included, "beef, pork or lamb as main dish", "hamburger", and "beef, pork or lamb as a sandwich or mixed dish"	processed red meat	Processed red meat included "bacon", "hot dogs", and "sausage, salami, bologna and other processed red meat"
Peng2021	none	none	Red meat	Red meat consisted of pork, beef and lamb	none	none
Zelber-Sagi2018	Total red meat	Total red meat was composed of: beef steak or roast, beef internal organs, fried beef patties, lamb and pork	none	none	processed meat	Processed meat included: hamburger, salami, pastrami, sausages, processed schnitzel and canned meat

Hashemian2020	Total red meat	total red meat intake including unprocessed meat (lamb, beef, and hamburger), organ meat (liver, kidney, gizzard, and heart), and processed meat (sausage and deli meat)	unprocessed red meat	unprocessed meat (lamb, beef, and hamburger)	none	none
Zhou2019	none	none	Red meat	Red meat intake was defined as the total amount of pork, beef and mutton	none	none
Tutunchi2021	Red and processed meat	Red and processed meat: Beef, lamb, camel, sausages, hamburger, processed meats, organ meats	none	none	none	none
Rahimi-Sakak2022	none	none	Red meat	red meat (beef or lamb or ground red meat)	processed meat	processed meat (sausages or burgers)
Honarvar2017	red meat	none	none	none	none	none

Table S2. Quality of the studies included in the meta-analysis according to Newcastle-Ottawa Scale.

**Risk Bias Assessment using Newcastle-Ottawa Scale for cross-sectional studies**								
**Study**	**selection**				**Comparability based on design and analysis**	**outcome**		**Total score**
	**Representativeness of the samples**	**Sample size**	**Non-respondents**	**Ascertainment of red meat eating**		**Assessment of outcome**	**Statistical test**	
Charatcharoenwitthaya2021	0	0	0	0	1	0	1	2
Peng2021	0	0	1	2	0	1	1	5

Zelber-Sagi2018	0	0	0	2	2	1	1	6
Zhou2019	1	0	1	1	1	1	1	6
Honarvar2017	1	1	1	2	1	0	1	7

Risk Bias Assessment using Newcastle-Ottawa Scale for cohort studies

**Study**	**Selection**				**Comparability of cohorts on the basis of the design or analysis controlled for confounders**	**Outcome**			**Total score**
	**Representativeness of the exposed cohort**	**Selection of the non-exposed cohort**	**Ascertainment of exposure**	**Demonstration that outcome of interest was not present at start of study**		**Assessment of outcome**	**Was follow-up long enough for outcomes to occur**	**Adequacy of follow-up**	
Kim2021	1	1	1	1	2	0	1	1	8
Hashemian2020	1	1	1	0	2	0	1	1	7
Noureddin2020	1	1	1	1	2	1	1	1	9

Risk Bias Assessment using Newcastle-Ottawa Scale for case-control studies

**Study**	**Selection**				**Comparability of cases and controls based on the design or analysis**	**Outcome**			**Total score**
	**Is the case definition adequate?**	**Representativeness of the cases**	**Selection of controls**	**Definition of controls**		**Assessment of exposure**	**Same method of ascertainment for cases and controls**	**Non-response rate**	
Tutunchi2021	1	0	0	1	1	1	1	0	5
Rahimi-Sakak2022	1	0	0	1	1	1	0	1	5

Table S3. Results of publication bias

	Egger's test	before trim-and-fill			after trim-and-fill		
		**point estimation**	**interval estimation**	**study number**	**point estimation**	**interval estimation**	**study number**
red meat	0.008	1.267	(1.073, 1.496)	8	0.994	(0.863, 1.144)	13
unprocessed red meat	0.100	1.279	(1.053, 1.552）	6	1.129	(0.909, 1.401)	8
processed red meat	0.298	1.195	(1.043, 1.370)	7	1.136	(0.965, 1.339)	9
